# Sex difference in the incidence of cardia and non-cardia gastric cancer in the United States, 1992–2014

**DOI:** 10.1186/s12876-020-01551-1

**Published:** 2020-12-11

**Authors:** Qiang Yao, Xiaona Qi, Shao-Hua Xie

**Affiliations:** 1 Department of Ultrasound Intervention, Harbin Medical University Cancer Hospital, Harbin, China; 2 Heilongjiang Provincial Academy of Medical Sciences, Harbin, China; 3 Nursing Department, Harbin Medical University Cancer Hospital, Harbin, 150081 Heilongjiang Province China; 4 Upper Gastrointestinal Surgery, Department of Molecular medicine and Surgery, Karolinska Institutet, Karolinska University Hospital, Stockholm, Sweden

**Keywords:** Stomach neoplasms, Incidence, Sex difference, Sex hormones, Etiology, SEER

## Abstract

**Background:**

Gastric cancer is more common in men than in women, but underlying reasons have not been completely understood. This study aimed to assess patterns of the sex difference in the incidence of gastric cancer in the United States.

**Methods:**

Using data from 13 cancer registries in the Surveillance, Epidemiology, and End Results Program, we analyzed the age-specific sex difference in the incidence of gastric cancer by ethnicity, anatomic site and histological type in the United States during 1992–2014. We assessed the temporal trends in the sex differences in the incidence of gastric cancer during the study period.

**Results:**

The male-to-female incidence ratio of cardia cancer increased with age until peaking at ages 55–69 years and decreased thereafter, while the ratio for non-cardia gastric cancer increased with age before ages < 60 years and remained stable onwards. The age-specific patterns in the sex difference of gastric cancer incidence varied between intestinal and diffuse histological types. The sex difference in the incidence of cardia cancer remained relatively stable except for that the absolute difference between the sexes in whites decreased on average by 0.8% per year from 1992 to 2014. The absolute incidence difference between the sexes in non-cardia gastric cancer decreased over time in whites, blacks, and Asian and Pacific islanders by approximately 4% per year. The male-to-female incidence ratio of non-cardia gastric cancer decreased over time in whites and blacks, but remained relatively stable in Asian and Pacific islanders.

**Conclusions:**

Both extrinsic and intrinsic factors may have contributed to the sex difference in gastric cancer. Sex hormones may play a role in the development of cardia cancer and intestinal type of gastric cancer.

## Background

Gastric cancer is the fifth most common cancer with an estimated nearly one million new cases occurring globally each year [1]. Gastric cancer varies greatly in epidemiology and risk factors across anatomic subsite and histological types [2, 3]. Cardia and non-cardia gastric cancer share some risk factors, such as tobacco smoking, and family history, and radiation [2]. *Helicobacter pylori* (*H. pylori*) infection is a major risk factor for non-cardia gastric cancer, but it does not increase the risk of cardia gastric cancer [2, 3]. On the other hand, obesity and gastroesophageal reflux disease have been consistently associated with increased risk of cardia gastric cancer but not non-cardia gastric cancer [2, 3]. The majority of gastric cancers are adenocarcinomas, which can be stratified as diffuse or intestinal subtypes. These two subtypes also differ in epidemiological features [4].

Both cardia and non-cardia gastric cancers have higher incidence in men than in women, and the sex difference is greater in cardia cancer [5]. The sex difference in gastric cancer may be partially explained by a higher exposure prevalence of certain risk factors, e.g., tobacco smoking and *H. pylori* infection, in men than in women. It has been hypothesized that sex hormones may play a role in the development of gastric cancer, i.e. female sex hormones protecting gastric cancer and/or male sex hormones increasing the risk [6]. However, existing epidemiological evidence remains limited and inconclusive [2, 3].

Using incidence data from the Surveillance, Epidemiology, and End Results (SEER) Program, we analyzed the sex difference in the incidence of gastric cancer by anatomic site, i.e. cardia and non-cardia gastric cancers, in the United States during a period of over 20 years. We aimed to provide insightful etiological clues from these descriptive analyses, particularly the possible variations by age, ethnicity and histological type.

## Methods

### Data sources

We extracted data on the number of newly diagnosed cases of gastric cardia cancer (topography code C16.0 according to the International Classification of Diseases for Oncology, 3rd edition [ICD-O-3]) and non-cardia gastric cancer (ICD-O-3 codes C16.1-C16.6) from the November 2016 submission of the SEER 13 registries database using the SEER*Stat program version 8.3.4 [7]. We also extracted data on corresponding population sizes from the SEER program. The SEER 13 database includes data from the 13 cancer registries across the United States during 1992 to 2014. These registries are cancer registries in Atlanta, Connecticut, Detroit, Hawaii, Iowa, New Mexico, San Francisco-Oakland, Seattle-Puget Sound, Utah, Los Angeles, and San Jose-Monterey, and Rural Georgia and the Alaska Native Tumor Registry [7].

### Statistical analysis

All gastric cancer patients were divided into 5-year groups starting from ages 30–34 years, and all patients younger than 30 years or older than 85 years were categorized into separate groups. Sex- and age group-specific incidence rates were computed for cardia and non-cardia gastric cancers separately by dividing the number of cases by the corresponding population size. We calculated the sex-specific age-standardized incidence rates using the direct method with the 2000 United States Standard Population as reference [8]. The male-to-female incidence ratios were calculated by dividing the age-standardized rates in males by those in females. We estimated the corresponding 95% confidence intervals [CI] in the incidence ratios between the sexes based on a log-normal distribution assumption. We also stratified the analysis for the three major ethnic groups (whites, blacks, and Asian and Pacific islanders) and for the two histological subtypes (intestinal and diffuse subtypes).

We assessed the temporal trends in the sex difference in cardia and non-cardia gastric cancers by ethnic group over the study period. Two indicators, male-to-female rate difference and incidence ratio, were used to measure the sex difference on the absolute and relative scales, respectively. Rate difference is the absolute difference between incidence rate in males and the rate in women and it is equal to 0 in the absence of sex disparity. Rate ratio, as described above, is a disparity measure on the relative scale, and a value of 1 corresponds to no disparity [9, 10]. We plotted the annual estimates of these measures of sex difference in the incidence against the calendar years. We further performed log-linear regressions to estimate the average annual percent change (APC) in each disparity measure under the assumption that this measure changed at a constant percentage ever year over the study period [11].

All statistical analyses were performed using the statistical software SAS version 9.4 (SAS Institute, Cary, NC) except for estimating the APC in the sex difference over time which was performed by the Joinpoint Regression Program version 4.5.0.1 developed by the National Institute of Cancer of the United States [12]. All *P* values are two-sided, and we considered a *P* value less than 0.05 as statistically significant.

The analyses were solely based on publicly available data of population sizes and aggregate number of cancer cases and as such, ethical approval was not deemed to be necessary.

## Results

This study included a total of 18,997 new cases of cardia cancer (14,614 males and 4383 females) and 38,537 new cases of non-cardia gastric cancer (21,134 males and 17,403 females), with the male-to-female ratios in age-standardized incidence rate of 4.2 for cardia cancer and 1.6 for non-cardia gastric cancer.

Table 1 presents the sex- and age-specific incidence rates of cardia and non-cardia gastric cancers, as well as the male-to-female ratios in the incidence. The male-to-female ratio in the incidence of cardia cancer was 1.9 at ages below 30 years, increased with age until peaking at ages 50–69 years (ranging from 4.7 to 5.1), and decreased with age thereafter. The sex ratio for non-cardia gastric cancer was lower than 1 for ages below 35 years (0.8), increased with age from ages 35–39 years before ages < 60 years and remained stable onwards.

**Table 1:** Age-Specific Incidence Rate (1/100,000 person-years) of Cardia and Non-Cardia Gastric Cancer by Sex and Male-to-Female Incidence Ratios in the United States, 1992–2014

Age, years			Cardia					Non-cardia		
	Male		Female		M/F ratio (95% CI)	Male		Female		M/F ratio (95% CI)
	Number	Rate	Number	Rate		Number	Rate	Number	Rate	
< 30	57	0.03	28	0.01	1.9 (1.2, 3.1)	123	0.1	150	0.1	0.8 (0.6, 1.0)
30–34	100	0.3	37	0.1	2.6 (1.8, 3.9)	178	0.5	207	0.6	0.8 (0.7, 1.0)
35–39	201	0.6	64	0.2	3.1 (2.4, 4.1)	359	1.0	343	1.0	1.0 (0.9, 1.2)
40–44	344	1.0	102	0.3	3.4 (2.7, 4.2)	609	1.8	560	1.6	1.1 (1.0, 1.2)
45–49	686	2.2	157	0.5	4.5 (3.8, 5.3)	961	3.0	741	2.3	1.3 (1.2, 1.5)
50–54	1098	3.9	269	0.9	4.2 (3.7, 4.9)	1278	4.6	936	3.2	1.4 (1.3, 1.5)
55–59	1593	6.9	336	1.4	5.0 (4.5, 5.7)	1654	7.2	1208	4.9	1.5 (1.3, 1.6)
60–64	1921	10.5	446	2.2	4.7 (4.3, 5.2)	2046	11.2	1342	6.7	1.7 (1.6, 1.8)
65–69	2183	15.3	497	3.0	5.1 (4.6, 5.6)	2670	18.7	1804	10.9	1.7 (1.6, 1.8)
70–74	2136	19.1	599	4.3	4.4 (4.1, 4.9)	3178	28.4	2161	15.5	1.8 (1.7, 1.9)
75–79	2026	23.7	616	5.2	4.5 (4.1, 5.0)	3113	36.5	2542	21.6	1.7 (1.6, 1.8)
80–84	1364	23.8	615	6.8	3.5 (3.2, 3.9)	2638	46.1	2487	27.4	1.7 (1.6, 1.8)
85+	905	21.1	618	6.7	3.2 (2.9, 3.5)	2327	54.2	2922	31.5	1.7 (1.6, 1.8)
All	14,614	3.3	4384	1.0	3.4 (3.3, 3.5)	21,134	4.7	17,403	3.8	1.2 (1.2, 1.3)
Age-standardized rate ^*^		3.8		0.9	4.2 (4.1, 4.4)		5.8		3.6	1.6 (1.6, 1.6)

*CI* confidence interval

^*^ Using 2000 United States Standard Population as reference

**Fig. 1:**
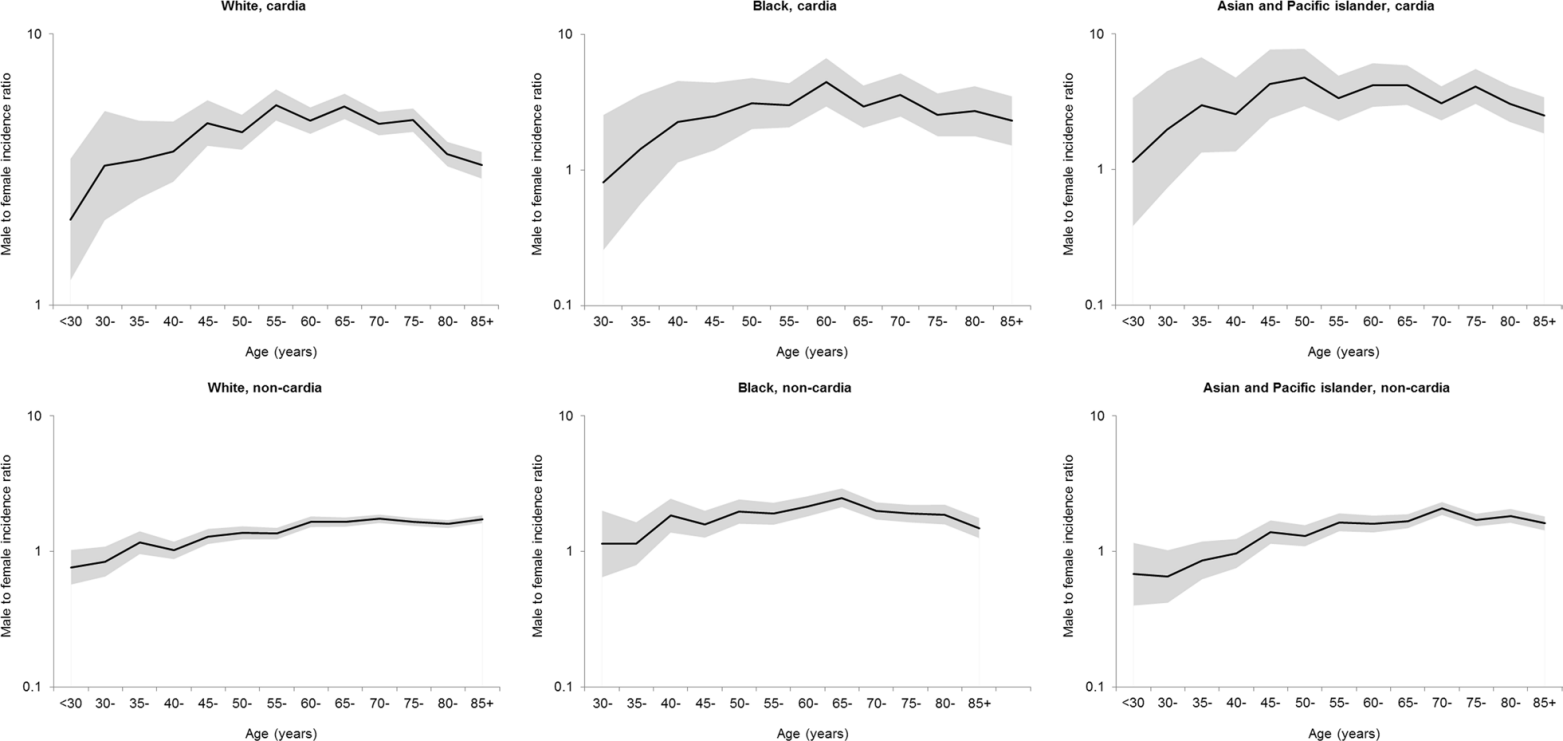
Age-specific male-to-female ratio in the incidence of cardia and non-cardia gastric cancer by ethnicity in the United States, 1992–2014

**Fig. 2:**
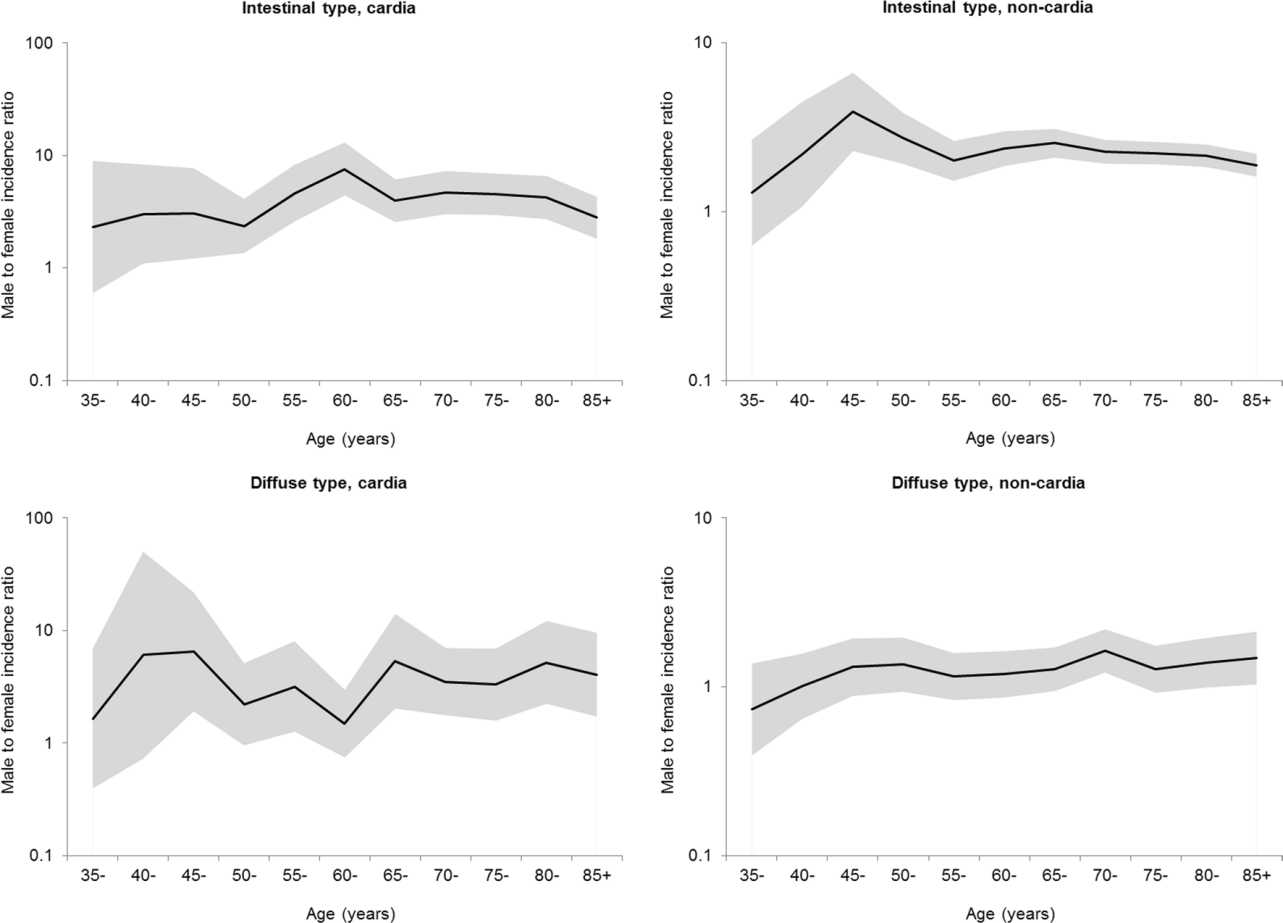
Age-specific male-to-female ratio in the incidence of intestinal and diffuse types of cancer by anatomic site in the United States, 1992–2014

The male-to-female ratios in the incidence of cardia and non-cardia gastric showed relatively similar patters across ethnic groups (Fig. 1). However, the ratio for non-cardia cancer in blacks was highest at ages 65–69 years and slightly decreased thereafter, whereas it generally increased with age until the ages 70–74 years and remained generally stable thereafter in whites and in Asian and Pacific islanders. The age-specific sex ratio curves displayed distinct patterns between the intestinal and diffuse histological subtypes (Fig. 2). Minor peaks at ages 60–64 years for cardia cancer and at 45–49 years for non-cardia cancer were observed in the age-specific sex ratio curves for intestinal type. In contrast, the sex ratio in the incidence of diffuse type gastric cancer remained relatively stable across age groups.

**Fig. 3:**
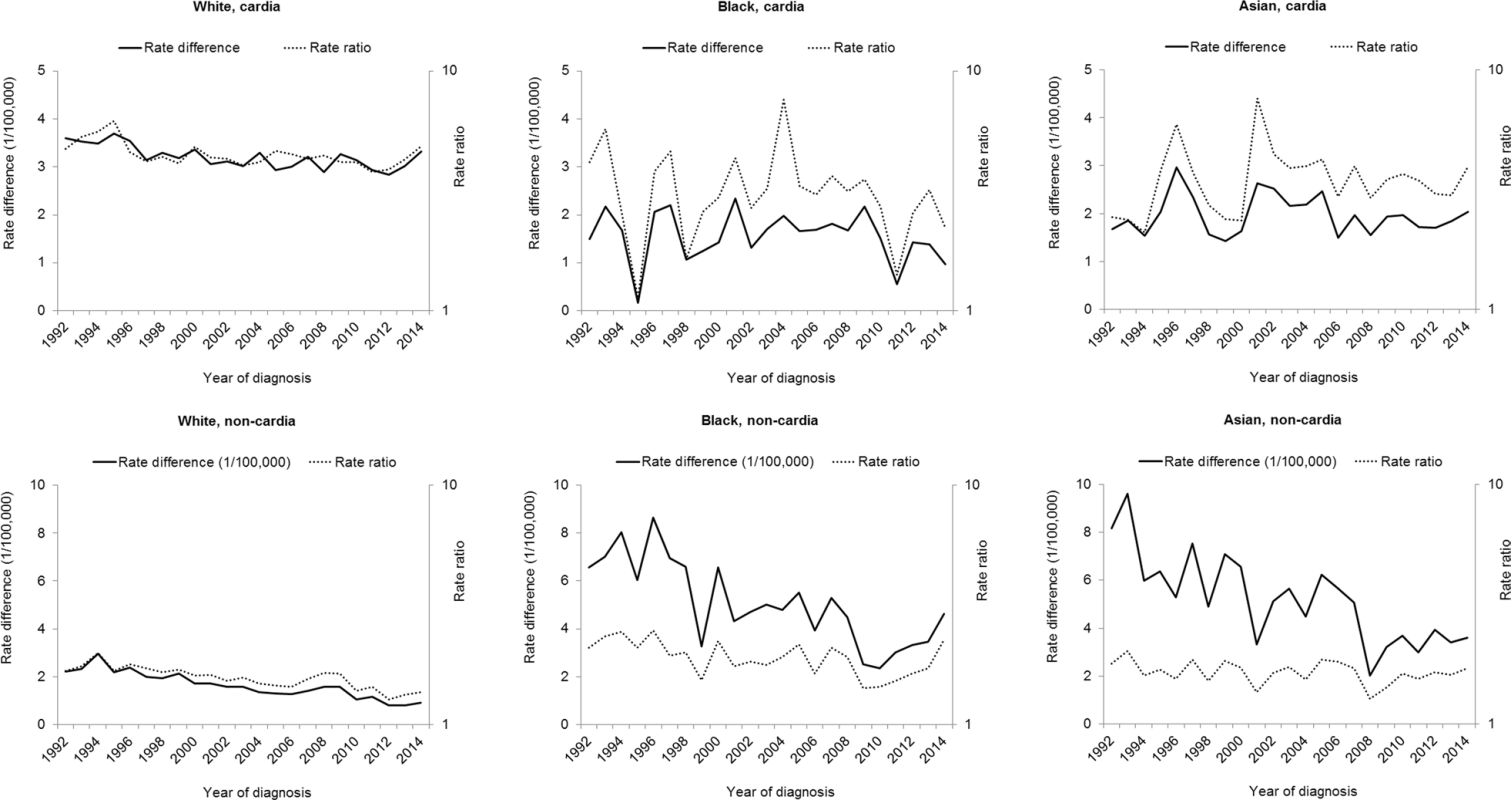
Annual male-to-female difference in the incidence of cardia and non-cardia gastric cancer by ethnicity in the United States, 1992–2014

As shown in Fig. 3, the sex difference in the incidence of cardia cancer remained relatively stable over time in all the three ethnic groups. However, the absolute difference in the incidence rate of cardia cancer between the sexes decreased on average by 0.8% (95% CI 0.4–1.1%) per year from 1992 to 2014 in whites (Table 2). The absolute incidence difference between the sexes in non-cardia gastric cancer decreased over time in whites, blacks, and Asian and Pacific islanders by approximately 4% per year. However, The male-to-female incidence ratio of non-cardia gastric cancer decreased over time in whites and blacks, but remained relatively stable in Asian and Pacific islanders (Fig. 3 and Table 2). Seemingly increased rate differences and rate ratios toward the end of the study period, i.e. since 2010 were observed for non-cardia gastric cancer in blacks and Asian and Pacific islanders (Fig. 3).

**Table 2:** Annual percent change in the male-to-female difference in the incidence rate of gastric cancer by anatomic site and ethnic group in the United States, 1992–2014

Site	Ethnicity	Rate difference		Rate ratio	
		APC	95% CI	APC	95% CI
Cardia	White	−0.8	−1.1 to −0.4	−0.5	−3.6 to 2.7
	Black	0.1	−3.7 to 4.0	−1.0	−3.7 to 1.9
	Asian and Pacific islander	−0.2	−1.5 to 1.2	0.8	−1.2 to 2.9
Non-cardia	White	−4.1	−7.4 to −0.6	−1.3	−1.7 to − 0.9
	Black	−4.0	−5.5 to −2.4	− 1.3	−2.2 to − 0.4
	Asian and Pacific islander	− 4.1	− 5.6 to − 2.5	− 0.5	− 1.2 to 0.2

*APC* annual percent change, *CI* confidence interval

## Discussion

This study provides a comprehensive analysis of the sex difference in the incidence of gastric cancer by anatomic site in the United States during a period of over 20 years. Our results confirmed that gastric cancer had been more common in men than in women, and such sex difference was more striking for cardia gastric cancer. We observed distinct age-specific patterns in the sex difference in incidence between cardia and non-cardia gastric cancers, and between intestinal and diffuse histological subtypes. The sex difference in the incidence of cardia cancer remained relatively stable during the period 1992–2014, whereas that for non-cardia gastric cancer generally decreased over time.

Analyzing the age-specific patterns of the sex difference in incidence may provide etiological clues on risk factors with age-dependent associations with gastric cancer risk. Particularly, if estrogens protect against the development of gastric cancer in women as hypothesized, the protective effect may be stronger before the menopausal ages than at postmenopausal ages, and thus, the male-to-female incidence ratio may decline accordingly after menopausal ages [13–16]. Our findings indicate an increasing male-to-female ratio in the incidence of cardia gastric cancer and a declined which was not observed for non-cardia gastric cancer. Moreover, a declined sex ratio in incidence was also observed for intestinal type rather than diffuse type of gastric cancer. Therefore, estrogens are more likely to protect against cardia cancer and intestinal type of gastric cancer rather than non-cardia gastric cancer or diffuse type of gastric cancer. Such findings are consistent with previous studies [13, 16]. The lack of decrease in the sex difference in the incidence of non-cardia gastric cancer over age indicates that risk factors other than sex hormonal exposures may be more relevant, particularly those in early life, e.g. *H. pylori* infection.

Gastric cardia cancer, mostly adenocarcinoma of intestinal type, is divergent from non-cardia gastric cancer but similar to esophageal adenocarcinoma in etiology. The striking male predominance in adenocarcinoma of the esophagus and gastric cardia cannot be explained by the two major risk factors, i.e. reflux and obesity, given the similar exposure prevalence and strengths of association with the risk of this cancer between the sexes, but abdominal obesity, the typical male-type adiposity, may partially explain [17, 18]. Tobacco smoking is a moderate risk factor and may contribute to such sex difference to a limited extent [17, 18]. It has been hypothesized that female sex hormones may protect against the development of this cancer, existing epidemiological evidence in humans however remains inconclusive [17]. A previous pooled analysis of three case-control studies in Western populations has revealed an inverse association between breastfeeding and the risk of adenocarcinoma of the esophagus and gastric cardia [19]. Chronic inflammation caused by gastric juice associated with long-standing reflux is an essential mechanism in the carcinogenesis of gastric cardia cancer [17]. The potential protective effect from female sex hormones may be explained by their anti-inflammation effect. Other possible mechanisms may include adaptation of cell cycle and inducing apoptosis malignant cells through estrogen receptor ligands [17, 20, 21].

Assessing the temporal trends in the sex difference of gastric cancer incidence also have useful implications. Any temporal changes in the sex difference reflect the contributions from environmental/extrinsic risk factors with changing prevalence differences between the sexes over time, while a stable sex difference indicate the role of intrinsic exposures or environmental risk factors with stable sex difference in prevalence. The decreasing sex difference in the incidence of non-cardia gastric cancer may be explained by the decreasing prevalence of *H. pylori* infection, particularly in men with historically higher prevalence [22, 23]. The decline in smoking prevalence in the United States, which is more rapid in men than in women, may have also contributed the decreased sex difference in non-cardia gastric cancer [24, 25]. However, this was not clearly reflected in the sex difference in cardia cancer. In addition, we have noted seemingly increased sex difference in the incidence of non-cardia gastric cancer since 2010 in blacks and Asian and Pacific islanders, which warrants confirmation through continued monitoring and investigations.

To our knowledge, this study is the most updated and comprehensive analysis of the age-specific sex difference in the incidence of gastric cancer by anatomic site, histological type, and ethnic group in the United States. This is also the first to quantitatively assess the time trends of the sex difference in the incidence of gastric cancer with disparity measures on both absolute and relative scales. Moreover, the SEER data with good quality have ensured the reliability of our findings. However, several limitations of the present study warrant some discussion. First, data used in this study only covered 13 cancer registries in the United States, and thus, may not be representative for the total population on a nationwide scale. Second, given the limited number of cases, the estimates for some subgroup analyses, e.g. cardia cancer in blacks or Asian and Pacific islanders, were lack of precision, and thus, role of chance should be considered when interpreting these results. Third, for the same reason, we did not separate whites into Hispanic and non-Hispanic groups despite the differential incidence in cardia and non-cardia gastric cancer in these two groups, i.e. higher incidence of cardia gastric cancer but lower incidence of non-cardia gastric cancer in non-Hispanic whites than in Hispanic whites [26]. Forth, intestinal and diffuse subtypes of adenocarcinomas were largely underreported in the SEER data, which resulted in low precision in analysis for these subtypes. However, the underreporting would have affected both sexes in an equal manner, which might not have biased male-to-female incidence ratios to a great degree. Finally, this is an ecological analysis without information on risk factors at the individual level, all speculated explanations for the sex difference in the risk of gastric cancer remain to be confirmed in analytic epidemiological studies.

## Conclusions

In summary, this study provides an updated and comprehensive analysis of the sex difference in the incidence of gastric cancer during a period of over 20 years in the United States. We observed divergent age-dependent patterns in the sex difference in the incidence between cardia and non-cardia gastric cancer and across histological subtypes. Our findings suggest a possibly protective role of female sex hormones against cardia cancer and intestinal type of gastric cancer. The results also demonstrate a narrowing sex difference in the incidence of non-cardia cancer. More etiological studies are needed to better understand the reasons for the sex difference in gastric cancer, which could provide evidence supporting prevention of this cancer in both sexes.

## Data Availability

The data used in this study are publicly available from the SEER program.

## Abbreviations

APC: annual percent change
CI: confidence interval
*H. pylori*: *Helicobacter pylori*
ICD-O-3: International Classification of Diseases for Oncology, 3rd edition
SEER: the Surveillance, Epidemiology, and End Results Program
